# Accidental Wounds of the Female Bladder

**Published:** 1900-01

**Authors:** 


### ACCIDENTAL WOUNDS OF THE FEMALE BLADDER.

FREDERICK HOLME WIGGIN, M. D., New York, (*Jour. Am. Med. Ass'n*, September 9, 1899,) says: Accidental opening of the bladder has, for many years, been considered one of the most serious accidents that could occur in the course of the complicated work which gynecic surgeons are often called upon to perform. The following case is offered in illustration of this type of injury:

M. H., unmarried, aged 41, was admitted to the City Hospital, Blackwell's Island, N. Y., September 30, 1898, suffering from a large myoma, which sprung from the anterior uterine wall and extended above the umbilicus. On October 3d, the abdomen was opened, and the tumor, which weighed seventeen pounds, was drawn down through an incision six inches in length, freed from its attachments and removed, together with the body of the uterus amputated near the internal os. As hemorrhage was profuse it became necessary to remove the mass very rapidly, to accomplish which the anterior attachment of the tumor was clamped and cut, when it was discovered from the escape of urine that the bladder had been opened near the fundus.

The general cavity had previously been shut off with gauze pads and thoroughly irrigated, followed by the use of hydrozone in half strength, and this, in turn, by saline solution. The gauze pads were now changed, and the opening in the bladder, four inches in length, was closed by means of two layers of chromicised catgut sutures. The wound was then disinfected, and there being a large peritoneal flap, it was attached to the bladder and made to cover the line of sutures, thus making the bladder wound extraperitoneal. After further washing out of the abdominal cavity with hydrozone and the saline solution, the external wound was closed, without drainage, and the usual dressings applied. The patient being feeble it was not thought advisable to make a vesico-vaginal fistula to drain the bladder, but, instead, a self-retaining catheter was introduced. At the end of ten days, however, tumefaction occurred over the lower angle of the abdominal wound, and, on opening it, urine began to escape. A vesico-vaginal fistula was now made in order to afford adequate drainage. The sinus in the abdominal wall was curetted and, after being thoroughly disinfected with hydrozone, its walls were sutured. Soon afterward, the sinus having closed, the sutures which kept open the vesico-vaginal fistula were removed, and the latter closed quickly without any further operative interference.

Percival (*British Medical Journal*, 1897, Vol. I., p. 1282,) reports a case of ruptured bladder on which he had operated. It was closed by means of a double wall of Lembert silk sutures. The wound in the abdominal wall was closed, after the peritoneal cavity had been flushed out with boric acid solution and a large quantity of clots and urinous fluids had been removed. For a few days the patient did well, and then died from peritonitis. But the necropsy proved that the bladder wound had completely healed. It is the writer's opinion that had saline solution and hydrozone been used, instead of boric acid, and the abdominal wound been closed leaving saline solution in the peritoneal cavity the patient would probably have recovered.

---

### HOT WATER DRINKING.

THERE are four classes of persons (*Indiana Lancet*) who should not drink large quantities of hot water. These are as follows:

1. People who have irritability of the heart. Hot water will cause palpitation of the heart in such cases.
2. Persons with dilated stomachs.
3. Persons afflicted with "sour stomach."
4. Persons who have soreness of the stomach, or pain induced by light pressure.

These rules are not for those who take hot water simply to relieve thirst better than cold water, and for that purpose is not to be condemned. But hot water is an excitant, and, in cases in which irritation of the stomach exists, should be avoided.—*Nashville Jour. Med. and Surgery.*

---

TREATMENT OF PERTUSSIS.—November *Pediatrics* contains a review of the therapeutics of pertussis by Kaumheimer in which he reports bromoform as the most satisfactory agent in relieving the frequency and severity of the spasmodic seizures and shortening the duration of the disease. Dosage—one drop for each year of age, given three and four times a day. It is best given in mucilaginous or syrupy mixtures and should always be prescribed by drops, as the fluid dram contains 480 drops, while the dram by weight, 160 drops. The susceptibility of patients varying, many cases of intoxication are reported, one fatal.—*Chicago Clinic.*

---

### FOR PROSTATITIS.

℞—Ichthyol . . . . . . . . . . . . . . . . . . . . . . . . ʒi.

Ol. theobrom . . . . . . . . . . . . . . . . . . . . . . . . ʒvi.

M. Ft. suppos No. xii. Sig. Use one suppository at night and one in the morning after defecation. Under this treatment the pain, sense of pressure, etc., are said to decrease rapidly, and the hypertrophy to become sensibly diminished. —A. FREUDENBERG, *Med. News.*

